# Persistent Idiopathic Dentoalveolar Pain in a Patient With Implant Placement: A Case Report

**DOI:** 10.7759/cureus.64420

**Published:** 2024-07-12

**Authors:** Alhanouf A Alturki, Waleed Alibrahim

**Affiliations:** 1 Dentistry, Prince Sultan Military Medical City, Riyadh, SAU

**Keywords:** chronic idiopathic dentoalveolar pain, orofacial trauma, oro-facial pain, nortriptyline, dental implant

## Abstract

Persistent idiopathic dentoalveolar pain (PIDAP) is a type of disease that, despite affecting thousands of people globally, negatively impacts patients' quality of life because of its unknown cause. Notably, the disease has a high prevalence rate and is primarily prone to middle-aged and senior individuals. Efforts have been made to gain the understanding needed for the accurate diagnosis and prompt treatment of PIDAP cases. This case report discusses the challenges faced in diagnosing and managing PIDAP after dental implants.

The present study involved the case of a 62-year-old male patient, previously operated on for an implant at position #11, who suffered from chronic pain but no specific cause could be identified. We used an evaluation strategy to gain insights into the patient’s illness, including antibiotic treatment, crown replacement, and continued pain. We prescribed nortriptyline 10 mg, and there was an improvement. This finding suggests that nortriptyline 10 mg QHS eliminates chronic pain.

## Introduction

Persistent idiopathic dentoalveolar pain (PIDAP) is a clinical challenge due to its obscure etiology and consistent symptomatology. In most cases, PIDAP is a great concern among patients with a history of implant placement. Therefore, initiating an evidence-based care plan to optimize the quality of life (QoL) is vital. The existence of this mysterious PIDAP disease is a good indication that much work is required to make a complete diagnosis and implement logical prevention and curative methods.

PIDAP was previously known as atypical odontalgia or phantom tooth pain. It is a very destructive illness that thousands of people develop across the world. PIDAP features persistent pain in the joint region, whose causes have not been determined [1,2]. Therefore, this condition can be keenly felt in the patient’s quality of life. J. Coulter argued that the likelihood of developing PIDAP ranges from 2% to 4%, predominantly affecting middle-aged or older adults [3]. Such a condition is drawing certain focus lately, especially in the dental implantology field, where many studies look into cases of PIDAP following implant placement [4,5]. This trend is not confined to a specific demographic but encompasses individuals undergoing dental implantation, warranting meticulous attention to its implications.

Prior scholars conducted a lot of studies and were very close to solving the problem, but there are still many reasons why this issue is still not completely solved. Malacarne represented a major and elaborate review of PIDAP. This is an important risk factor that is difficult to diagnose and manage [6]. Jang JH compared the clinical features of PIDAP with those of inflammatory pain and, in doing so, gave valuable hints on the distinction between these two illnesses [7]. However, a gap in the literature exists regarding the prevalence and management of PIDAP in patients who have undergone dental implant placement. There is a need for further research to understand the incidence and risk factors as well as optimal treatment strategies for PIDAP in this patient population [4,5].

This case is crucial for a better understanding of the scope of orofacial and periodontal diseases. The aim of this study this case is to give healthcare providers the understanding needed for the accurate diagnosis and prompt treatment of PIDAP cases to promote their patients' quality of life. The potency of this project relies on its ability to optimize treatment guidelines as well as to reduce PIDAP manifestation in individuals.

## Case presentation

**Figure 1:**
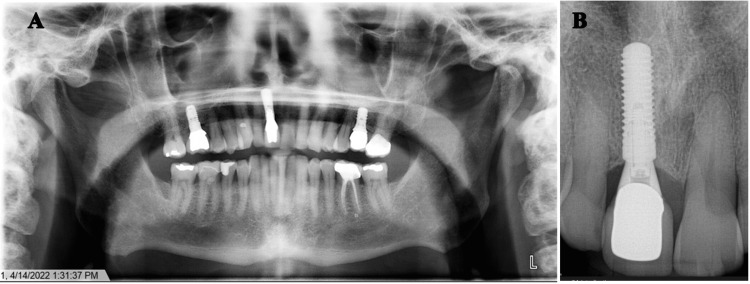
OPG and periapical radiograph A. OPG radiograph showing the implant on tooth #11 is particularly long, but it is not clear if it has any relation with the nasal cavity or the nasopalatine nerve. No destruction or invasion of the adjacent structures. B. Periapical radiograph showing that the implant on tooth #11 is particularly long, 12 mm in length, and has no major odontogenic findings. OPG: orthopantomogram

A 62-year-old male was referred with the chief complaint of dentoalveolar pain. He reported it to be present for two years. Around 2020, the patient had an implant placement in tooth #11, which appeared particularly long, 12 mm in length, without any complications noted (Figures 1A-1B). In 2022, he started to have pain in the gum around #11 spreading to #21 without any inciting event.

In 2022, he sought consultation with his periodontist who replaced the implant's crown and noticed a periodontal abscess in the area. It was treated with drainage, mechanical debridement, and antibiotic therapy; in addition, it was recommended to change the crown abutment.

**Figure 2:**
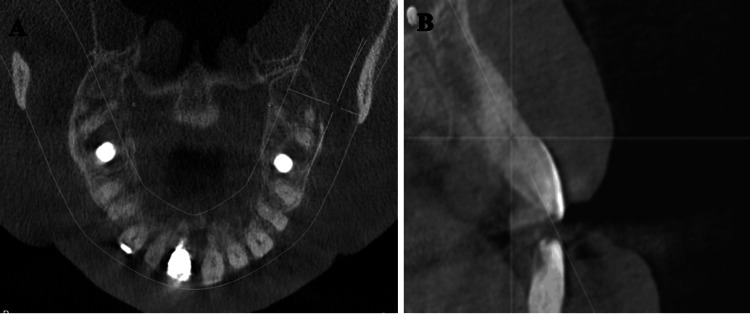
CT scan A. Axial and B. Lateral view of CT scan of the upper jaw, which showed unremarkable findings in bone volume and density

The patient followed these recommendations but the pain persisted unchanged. In April 2023, he had a CT scan (Figures 2A-2B), which showed unremarkable findings in bone volume and density of the upper jaw. In May 2023, he started to feel a sharp pain in tooth #21 then he sought a consultation with an endodontist who ruled out the odontogenic cause of the pain and referred him back to the periodontist.

The patient was previously diagnosed with seasonal hypertension, and his blood pressure had higher readings during winter. And he might be able to reduce the dosage when spring comes or stop taking the medication once summer rolls around. Also, he reported a history of spinal stenosis, which caused his back pain and stiff neck muscles as soon as he did the physical therapy sessions, his neck discomfort improved. The patient denied widespread body pain. He is otherwise healthy and denies having any other medical condition. However, he reports significant fatigue associated with his pain. His family medical history is noncontributory.

He was on the following medication: tamsulosin 0.8 mg QD, restasis 0.5% BID, diltiazem 180 mg QD, gabapentin 700 mg TID, lorazepam 1 mg QD, tramadol 50 mg QD, and denies currently being on any other medication or medical treatment.

Patient laboratory tests were within normal range, including complete blood count (CBC), prothrombin time (PT), activated partial thromboplastin time (aPTT), and general profile. The differential diagnosis included localized inflammation intracranial pathology, temporomandibular joint (TMJ) disorder, and trigeminal neuralgia.

The extraoral examination revealed facial skin asymmetry, normal articular without noises, there are no trigger points, maximum mouth opening of 45.6 (mm). And open his mouth without experiencing any pain or discomfort. The intraoral examination revealed linea alba in the buccal mucosa, normal tongue, and occlusal wear of teeth due to bruxism. 

The pain assessment developed gradually and went from intermittent and mild to constant and moderate. Soreness and occasional throbbing pain located deep in the alveolar bone around teeth #11 and #21. Pain randomly fluctuates in intensity from mild to moderate throughout the day. The patient always experiences increased pain when he lays down in the supine position, and pain gradually decreases after 10-15 minutes. Pain can wake the patient up from sleep. Caffeine consumption seems also to aggravate the pain. Pain improved when he is in an upright position, with topical application of benzocaine gel and with distraction. He denied any sensitivity to light or sounds and nausea or vomiting. Pain does not limit the patient's physical activities. The patient reported occasional tingling and "numb-like" sensation in the painful area (Table 1).

**Table 1:** Pain assessment, jaw evaluation, and orofacial examination findings

Pain assessment, jaw evaluation, and orofacial examination findings			
Pain characteristics and patterns			
Spontaneous/Provoked	Pain description	Worsening factors	Alleviation factors
Spontaneous	Gradual and became from intermittent and mild to constant and moderate, tingling and "numb-like"	Rest, caffeine	Cold, warm
Jaw evaluation			
Articular noises	Number of trigger points	Max mouth opening (mm)	Pain at maximum mouth opening
None	None	45.6	0
Orofacial examination			
Facial skin	Oral mucosa	Tongue	Teeth
Assimetry	Linea alba	Normal	Teeth worn by bruxism

The patient has been experiencing pain in the right side of the face once every six months, he denied clenching or pain with chewing and talking. He initially sought consultation with his general dentist shortly after his symptoms began, where he was evaluated and instructed to perform jaw stretching exercises daily at home. In addition, heat therapy consists of hot compresses applied periodically to the affected areas. He reported that his symptoms slightly improved with this regimen but added that he continued experiencing pain.

Regarding the headaches, he states that they began shortly after his facial pain and adds that they seemed to be triggered by stressful events. He points to the sinus area bilaterally and describes them as dull/pressure-like, sometimes throbbing in quality, and moderate intensity. They take place once or twice a week, usually upon awakening in the morning, and last no longer than three to four hours. He sought consultation with his ENT shortly after his symptoms began, where he did a sinus endoscopy which improved his symptoms. They are not incapacitating nor aggravated by routine physical activity, and he is usually able to continue with his daily activities when he gets them. He denies experiencing any visual, motor, or sensory disturbances nor any phonophobia, nausea, or vomiting. He also denies experiencing autonomic symptoms such as lacrimation, rhinorrhea, or scleral injection.

A few weeks later, at the first follow-up visit, the patient stated that he was following the self-management plan, including resting, facial massage, hot and cold packs, exercise, stretching, and the use of vitamin supplements. He was also using the prescribed medications and felt that this approach was somewhat helpful in controlling his symptoms with his headaches even presenting less frequently.

At the second follow-up office visit, the patient stated that he noticed a worsening in his symptoms after completing the pharmacotherapy. He reported an increase in his nighttime bruxism along with jaw pain/stiffness and headache mostly in the morning upon awakening. He was using the self-management program, as he thought they were very helpful in controlling his symptoms. The patient was prescribed nortriptyline 10 mg to be taken at bedtime. Nortriptyline has excellent effectiveness in the treatment of chronic pain because it works by reducing the amount of pain messages that arrive in the brain. In addition to that a course of physical therapy and oral orthotic stabilization appliance.

At the third follow-up office visit, the patient reported a significant improvement in his symptoms with occasional mild jaw pain and very infrequent headaches. He had been evaluated by a behavioral pain psychologist and was practicing meditation regularly, which he reported being very helpful along with the prescribed medication. Overall, he was doing well and felt that his symptoms had significantly improved since his initial evaluation with us. The patient was very pleased with the outcome of the treatment plan. At this point, the patient was discharged from active care and was told to follow up with us as needed.

## Discussion

PIDAP remains a condition that causes much difficulty in clinical practice, especially after implant surgery. The present case report describes the challenges of diagnosing and treating PIDAP, stressing the importance of professional cooperation. The patient’s case of developing chronic pain after implanting a reconstructed maxillary and mandibular dentition underlining PIDAP subjectivity is a good example of how PIDAP must be considered before giving another differential diagnosis when conventional courses of action do not help alleviate pain. Thus, an increased risk of PIDAP after dental implant surgeries, indicating an invasive dental procedure’s possible association with the development of this disorder [8]. The clinical implication here is clear: dental practitioners should highly suspect PIDAP in patients with pain that persists after implantation, which imaging studies and clinical examination cannot explain.

Furthermore, the case also explains that other diagnostic tools cannot diagnose the root cause of PIDAP. Consequently, despite comprehensive radiographic analysis, including a CT scan, no findings would indicate an anatomical basis for the patient’s complaints. This diagnostic challenge is one of the features of PIDAP and has underlined the necessity of superior diagnostic approaches. Also, studies have revealed the possibility of using functional MRI to pinpoint the neural biomarkers of PIDAP, which may improve PIDAP diagnosis in the future [2]. In clinical practice, this means that when faced with patients with chronic pain without definite organic pathology, a practitioner should refer the patient for advanced neuroimaging that might elucidate coordination, sensory, and motor (CSM) functions in pain processing and maintenance.

The treatment approach adopted in this case, particularly the use of nortriptyline, reflects current best practices in managing neuropathic pain conditions. This tricyclic antidepressant had a positive response, which supports the neurological etiology of PIDAP and the feasibility of centrally-acting medications. However, such medications should be considered as the components of the integrated therapy strategy. Thus, physical therapy, orthotic appliances, and psychological treatment methods applied in this case reflect an approach based on the biopsychosocial model in pain management. Clinicians should know that the treatment of PIDAP almost always entails a complex process, which may be challenging during care [3]. This includes the recognition of physical manifestations of the condition as well as the patient’s psychological and social features arising from the chronic pain.

Moreover, the patient reported relief from facial massages, hot and cold application, and exercise contentment, which emphasizes the need for patient education and directions in chronic pain. Regardless, these non-pharmacological interventions can significantly alleviate symptoms and should be components of the treatment plan. In addition, the use of meditation and psychological support, in this case, is another example of the increasing awareness of the psychological factors in the experience of chronic pain. Clinicians may wish to prescribe such complementary and alternative medicine (CAM) therapies for patients as approaches to managing PIDAP, which is a heterogeneous condition, given the many ways in which people experience pain.

Finally, this case report has emphasized the chronic nature of PIDAP and the need for long-term follow-up and management. The fluctuation in symptoms over time, including periods of improvement followed by exacerbations, is characteristic of many chronic pain conditions. This pattern underscores the need for long-term management strategies with directional changes that are amenable to variations depending on the patient’s condition. It appears that clinicians should cooperate with the patients and be ready to continue supporting them and changing the management tactics. Furthermore, it is encouraging to note that through the above changes in treatment, the patient with PIDAP got a favorable outcome through medical and non-medical ways of handling the illness. It highlights the quality of life that a caring and holistic approach toward patients can achieve.

## Conclusions

Even though the cause of idiopathic dentoalveolar pain is unclear, despite the employment of medical diagnostic procedures, some techniques have been developed to manage this illness. In the present case, PIDAP resolved immediately after using nortriptyline 10 mg as a therapeutic agent. This therapeutic agent is essential because it is used as treatment rather than as a preventive and has shown positivity where standard treatments have been unresponsive. However, there is still a significant gap in managing and treating the condition. Therefore, there is a need for further studies to identify targeted approaches to the treatment of the disease.

## References

[1] Van der Cruyssen F, Van Tieghem L, Croonenborghs TM (2020). Orofacial quantitative sensory testing: current evidence and future perspectives. Eur J Pain.

[2] Cervantes-Chavarría AR (2022). Persistent idiopathic dentoalveolar pain. Literature review and clinical case treated with intraoral application of botulinum toxin. Odovtos-Int J Dent Sc.

[3] Coulter J, Nixdorf DR (2020). A review of persistent idiopathic dentoalveolar pain (formerly PDAP/atypical odontalgia). Oral Surg.

[4] Patel J, Nixon P (2018). Chronic idiopathic pain following implant placement in the anterior maxilla: a case series. Dent Update.

[5] Rodríguez-Lozano FJ, Sanchez-Pérez A, Moya-Villaescusa MJ, Rodríguez-Lozano A, Sáez-Yuguero MR (2010). Neuropathic orofacial pain after dental implant placement: review of the literature and case report. Oral Surg Oral Med Oral Pathol Oral Radiol Endod.

[6] Malacarne A, Spierings EL, Lu C, Maloney GE (2018). Persistent dentoalveolar pain disorder: a comprehensive review. J Endod.

[7] Jang JH, Chung JW (2022). Clinical features of the persistent idiopathic dentoalveolar pain compared with inflammatory dental pain. J Oral Med Pain.

[8] Sanner F, Sonntag D, Hambrock N, Zehnder M (2022). Patients with persistent idiopathic dentoalveolar pain in dental practice. Int Endod J.

